# Supplementary material for: The MBD7 complex promotes expression of methylated transgenes without significantly altering their methylation status
Source: eLife. 2017 Apr 28;6:e19893. doi: 10.7554/eLife.19893 (PMC5462541; doi:10.7554/eLife.19893)
Supplement: Figure 1—source data 1. — Clustal alignment numbered relative to YJ. Perfectly matched residues are marked with an asterisk (*) below the alignment. DOI: http://dx.doi.org/10.7554/eLife.19893.003 [file elife-19893-fig1-data1.pdf]

CLUSTAL 2.1 multiple sequence alignment

```
YJ              GTTTAAGCGTCAATTGATAGTTTAAACTGAAGGCGGGAAACGACAATCTGATCCAAGCTC 60
LUCH            GTTTAAGCGTCAATTGATAGTTTAAACTGAAGGCGGGAAACGACAATCTGATCCAAGCTC 60
                ************************************************************

YJ              AAGCTGCTCTAGCATTCGCCATTCAGGCTGCGCAACTGTTGGGAAGGGCGATCGGTGCGG 120
LUCH            AAGCTGCTCTAGCATTCGCCATTCAGGCTGCGCAACTGTTGGGAAGGGCGATCGGTGCGG 120
                ************************************************************

YJ              GCCTCTTCGCTATTACGCCAGCTGGCGAAAGGGGGATGTGCTGCAAGGCGATTAAGTTGG 180
LUCH            GCCTCTTCGCTATTACGCCAGCTGGCGAAAGGGGGATGTGCTGCAAGGCGATTAAGTTGG 180
                ************************************************************

YJ              GTAACGCCAGGGTTTTCCCAGTCACGACGTTGTAAAACGACGGCCAGTGCCAAGCTTGCA 240
LUCH            GTAACGCCAGGGTTTTCCCAGTCACGACGTTGTAAAACGACGGCCAGTGCCAAGCTTGCA 240
                ************************************************************

YJ              TGCCTGCAGGTCGACGGTATCGATAAGCTTGATATCGAATTCCTGCAGGTCAACATGGTG 300
LUCH            TGCCTGCAGGTCGACGGTATCGATAAGCTTGATATCGAATTCCTGCAGGTCAACATGGTG 300
                ************************************************************

YJ              GAGCACGACACACTTGTCTACTCCAAAAATATCAAAGATACAGTCTCAGAAGACCAAAGG 360
LUCH            GAGCACGACACACTTGTCTACTCCAAAAATATCAAAGATACAGTCTCAGAAGACCAAAGG 360
                ************************************************************

YJ              GCAATTGAGACTTTTCAACAAAGGGTAATATCCGGAAACCTCCTCGGATTCCATTGCCCA 420
LUCH            GCAATTGAGACTTTTCAACAAAGGGTAATATCCGGAAACCTCCTCGGATTCCATTGCCCA 420
                ************************************************************

YJ              GCTATCTGTCACTTTATTGTGAAGATAGTGGAAAAGGAAGGTGGCTCCTACAAATGCCAT 480
LUCH            GCTATCTGTCACTTTATTGTGAAGATAGTGGAAAAGGAAGGTGGCTCCTACAAATGCCAT 480
                ************************************************************

YJ              CATTGCGATAAAGGAAAGGCCATCGTTGAAGATGCCTCTGCCGACAGTGGTCCCAAAGAT 540
LUCH            CATTGCGATAAAGGAAAGGCCATCGTTGAAGATGCCTCTGCCGACAGTGGTCCCAAAGAT 540
                ************************************************************

YJ              GGACCCCCACCCACGAGGAGCATCGTGGAAAAAGAAGACGTTCCAACCACGTCTTCAAAG 600
LUCH            GGACCCCCACCCACGAGGAGCATCGTGGAAAAAGAAGACGTTCCAACCACGTCTTCAAAG 600
                ************************************************************

YJ              CAAGTGGATTGATGTGATAACGTGGTGGAGCACGACACACTTGTCTACTCCAAAAATATC 660
LUCH            CAAGTGGATTGATGTGATAACGTGGTGGAGCACGACACACTTGTCTACTCCAAAAATATC 660
                ************************************************************

YJ              AAAGATACAGTCTCAGAAGACCAAAGGGCAATTGAGACTTTTCAACAAAGGGTAATATCC 720
LUCH            AAAGATACAGTCTCAGAAGACCAAAGGGCAATTGAGACTTTTCAACAAAGGGTAATATCC 720
                ************************************************************

YJ              GGAAACCTCCTCGGATTCCATTGCCCAGCTATCTGTCACTTTATTGTGAAGATAGTGGAA 780
LUCH            GGAAACCTCCTCGGATTCCATTGCCCAGCTATCTGTCACTTTATTGTGAAGATAGTGGAA 780
                ************************************************************

YJ              AAGGAAGGTGGCTCCTACGAATGCCATCATTGCGATAAAGGAAAGGCCATCGTTGAAGAT 840
LUCH            AAGGAAGGTGGCTCCTACGAATGCCATCATTGCGATAAAGGAAAGGCCATCGTTGAAGAT 840
                ************************************************************

YJ              GCCTCTGCCGACAGTGGTCCCAAAGATGGACCCCCACCCACGAGGAGCATCGTGGAAAAA 900
```

```
LUCH            GCCTCTGCCGACAGTGGTCCCAAAGATGGACCCCCACCCACGAGGAGCATCGTGGAAAAA 900
                ************************************************************

YJ              GAAGACGTTCCAACCACGTCTTCAAAGCAAGTGGATTGATGTGATATCTCCACTGACGTA 960
LUCH            GAAGACGTTCCAACCACGTCTTCAAAGCAAGTGGATTGATGTGATATCTCCACTGACGTA 960
                ************************************************************

YJ              AGGGATGACGCACAATCCCACTATCCTTCGCAAGACCCTTCCTCTATATAAGGAAGTTCA 1020
LUCH            AGGGATGACGCACAATCCCACTATCCTTCGCAAGACCCTTCCTCTATATAAGGAAGTTCA 1020
                ************************************************************

YJ              TTTCATTTGGAGAGGACCTCGAGAATTCTCAACACAACATATACAAAACAAACGAATCTC 1080
LUCH            TTTCATTTGGAGAGGACCTCGAGAATTCTCAACACAACATATACAAAACAAACGAATCTC 1080
                ************************************************************

YJ              AAGCAATCAAGCATTCTACTTCTATTGCAGCAATTTAAATCATTTCTTTTAAAGCAAAAG 1140
LUCH            AAGCAATCAAGCATTCTACTTCTATTGCAGCAATTTAAATCATTTCTTTTAAAGCAAAAG 1140
                ************************************************************

YJ              CAATTTTCTGAAAATTTTCACCATTTACGAACGATAGCCATGGAAGACGCCAAAAACATA 1200
LUCH            CAATTTTCTGAAAATTTTCACCATTTACGAACGATAGCCATGGAAGACGCCAAAAACATA 1200
                ************************************************************

YJ              AAGAAAGGCCCGGCGCCATTCTATCCGCTGGAAGATGGAACCGCTGGAGAGCAACTGCAT 1260
LUCH            AAGAAAGGCCCGGCGCCATTCTATCCGCTGGAAGATGGAACCGCTGGAGAGCAACTGCAT 1260
                ************************************************************

YJ              AAGGCTATGAAGAGATACGCCCTGGTTCCTGGAACAATTGCTTTTACAGATGCACATATC 1320
LUCH            AAGGCTATGAAGAGATACGCCCTGGTTCCTGGAACAATTGCTTTTACAGATGCACATATC 1320
                ************************************************************

YJ              GAGGTGGACATCACTTACGCTGAGTACTTCGAAATGTCCGTTCGGTTGGCAGAAGCTATG 1380
LUCH            GAGGTGGACATCACTTACGCTGAGTACTTCGAAATGTCCGTTCGGTTGGCAGAAGCTATG 1380
                ************************************************************

YJ              AAACGATATGGGCTGAATACAAATCACAGAATCGTCGTATGCAGTGAAAACTCTCTTCAA 1440
LUCH            AAACGATATGGGCTGAATACAAATCACAGAATCGTCGTATGCAGTGAAAACTCTCTTCAA 1440
                ************************************************************

YJ              TTCTTTATGCCGGTGTTGGGCGCGTTATTTATCGGAGTTGCAGTTGCGCCCGCGAACGAC 1500
LUCH            TTCTTTATGCCGGTGTTGGGCGCGTTATTTATCGGAGTTGCAGTTGCGCCCGCGAACGAC 1500
                ************************************************************

YJ              ATTTATAATGAACGTGAATTGCTCAACAGTATGGGCATTTCGCAGCCTACCGTGGTGTTC 1560
LUCH            ATTTATAATGAACGTGAATTGCTCAACAGTATGGGCATTTCGCAGCCTACCGTGGTGTTC 1560
                ************************************************************

YJ              GTTTCCAAAAAGGGGTTGCAAAAAATTTTGAACGTGCAAAAAAAGCTCCCAATCATCCAA 1620
LUCH            GTTTCCAAAAAGGGGTTGCAAAAAATTTTGAACGTGCAAAAAAAGCTCCCAATCATCCAA 1620
                ************************************************************

YJ              AAAATTATTATCATGGATTCTAAAACGGATTACCAGGGATTTCAGTCGATGTACACGTTC 1680
LUCH            AAAATTATTATCATGGATTCTAAAACGGATTACCAGGGATTTCAGTCGATGTACACGTTC 1680
                ************************************************************

YJ              GTCACATCTCATCTACCTCCCGGTTTTAATGAATACGATTTTGTGCCAGAGTCCTTCGAT 1740
LUCH            GTCACATCTCATCTACCTCCCGGTTTTAATGAATACGATTTTGTGCCAGAGTCCTTCGAT 1740
                ************************************************************

YJ              AGGGACAAGACAATTGCACTGATCATGAACTCCTCTGGATCTACTGGTCTGCCTAAAGGT 1800
LUCH            AGGGACAAGACAATTGCACTGATCATGAACTCCTCTGGATCTACTGGTCTGCCTAAAGGT 1800
```

```
                    ************************************************************

YJ                  GTCGCTCTGCCTCATAGAACTGCCTGCGTGAGATTCTCGCATGCCAGAGATCCTATTTTT 1860
LUCH                GTCGCTCTGCCTCATAGAACTGCCTGCGTGAGATTCTCGCATGCCAGAGATCCTATTTTT 1860
                    ************************************************************

YJ                  GGCAATCAAATCATTCCGGATACTGCGATTTTAAGTGTTGTTCCATTCCATCACGGTTTT 1920
LUCH                GGCAATCAAATCATTCCGGATACTGCGATTTTAAGTGTTGTTCCATTCCATCACGGTTTT 1920
                    ************************************************************

YJ                  GGAATGTTTACTACACTCGGATATTTGATATGTGGATTTCGAGTCGTCTTAATGTATAGA 1980
LUCH                GGAATGTTTACTACACTCGGATATTTGATATGTGGATTTCGAGTCGTCTTAATGTATAGA 1980
                    ************************************************************

YJ                  TTTGAAGAAGAGCTGTTTCTGAGGAGCCTTCAGGATTACAAGATTCAAAGTGCGCTGCTG 2040
LUCH                TTTGAAGAAGAGCTGTTTCTGAGGAGCCTTCAGGATTACAAGATTCAAAGTGCGCTGCTG 2040
                    ************************************************************

YJ                  GTGCCAACCCTATTCTCCTTCTTCGCCAAAAGCACTCTGATTGACAAATACGATTTATCT 2100
LUCH                GTGCCAACCCTATTCTCCTTCTTCGCCAAAAGCACTCTGATTGACAAATACGATTTATCT 2100
                    ************************************************************

YJ                  AATTTACACGAAATTGCTTCTGGTGGCGCTCCCCTCTCTAAGGAAGTCGGGGAAGCGGTT 2160
LUCH                AATTTACACGAAATTGCTTCTGGTGGCGCTCCCCTCTCTAAGGAAGTCGGGGAAGCGGTT 2160
                    ************************************************************

YJ                  GCCAAGAGGTTCCATCTGCCAGGTATCAGGCAAGGATATGGGCTCACTGAGACTACATCA 2220
LUCH                GCCAAGAGGTTCCATCTGCCAGGTATCAGGCAAGGATATGGGCTCACTGAGACTACATCA 2220
                    ************************************************************

YJ                  GCTATTCTGATTACACCCGAGGGGGATGATAAACCGGGCGCGGTCGGTAAAGTTGTTCCA 2280
LUCH                GCTATTCTGATTACACCCGAGGGGGATGATAAACCGGGCGCGGTCGGTAAAGTTGTTCCA 2280
                    ************************************************************

YJ                  TTTTTTGAAGCGAAGGTTGTGGATCTGGATACCGGGAAAACGCTGGGCGTTAATCAAAGA 2340
LUCH                TTTTTTGAAGCGAAGGTTGTGGATCTGGATACCGGGAAAACGCTGGGCGTTAATCAAAGA 2340
                    ************************************************************

YJ                  GGCGAACTGTGTGTGAGAGGTCCTATGATTATGTCCGGTTATGTAAACAATCCGGAAGCG 2400
LUCH                GGCGAACTGTGTGTGAGAGGTCCTATGATTATGTCCGGTTATGTAAACAATCCGGAAGCG 2400
                    ************************************************************

YJ                  ACCAACGCCTTGATTGACAAGGATGGATGGCTACATTCTGGAGACATAGCTTACTGGGAC 2460
LUCH                ACCAACGCCTTGATTGACAAGGATGGATGGCTACATTCTGGAGACATAGCTTACTGGGAC 2460
                    ************************************************************

YJ                  GAAGACGAACACTTCTTCATCGTTGACCGCCTGAAGTCTCTGATTAAGTACAAAGGCTAT 2520
LUCH                GAAGACGAACACTTCTTCATCGTTGACCGCCTGAAGTCTCTGATTAAGTACAAAGGCTAT 2520
                    ************************************************************

YJ                  CAGGTGGCTCCCGCTGAATTGGAATCCATCTTGCTCCAACACCCCAACATCTTCGACGCA 2580
LUCH                CAGGTGGCTCCCGCTGAATTGGAATCCATCTTGCTCCAACACCCCAACATCTTCGACGCA 2580
                    ************************************************************

YJ                  GGTGTCGCAGGTCTTCCCGACGATGACGCCGGTGAACTTCCCGCCGCCGTTGTTGTTTTG 2640
LUCH                GGTGTCGCAGGTCTTCCCGACGATGACGCCGGTGAACTTCCCGCCGCCGTTGTTGTTTTG 2640
                    ************************************************************

YJ                  GAGCACGGAAAGACGATGACGGAAAAAGAGATCGTGGATTACGTCGCCAGTCAAGTAACA 2700
LUCH                GAGCACGGAAAGACGATGACGGAAAAAGAGATCGTGGATTACGTCGCCAGTCAAGTAACA 2700
                    ************************************************************
```

```
YJ      ACCGCGAAAAAGTTGCGCGGAGGAGTTGTGTTTGTGGACGAAGTACCGAAAGGTCTTACC 2760
LUCH    ACCGCGAAAAAGTTGCGCGGAGGAGTTGTGTTTGTGGACGAAGTACCGAAAGGTCTTACC 2760
        ************************************************************

YJ      GGAAAACTCGACGCAAGAAAAATCAGAGAGATCCTCATAAAGGCCAAGAAGGGCGGAAAG 2820
LUCH    GGAAAACTCGACGCAAGAAAAATCAGAGAGATCCTCATAAAGGCCAAGAAGGGCGGAAAG 2820
        ************************************************************

YJ      ATCGCCGTGTAATTCTAGAGATTCTCTGTGTAAGCG----AAAGTCTAGAGTCCGCAAAA 2876
LUCH    ATCGCCGTGGGATCCCACCGGTTTGATGGTCGGGCCTCGACGAACCAAGTGTTGACAAAT 2880
        ********* .** * * .*.**  .**   ..**     ..*. *:**:**  .****:

YJ      --------ATCACCAG---TCTCT-CTCTACAAATCTATCT-----------CTCTCTAT 2913
LUCH    GCTGCAGCATCATCAGGATTCTCTCCTCATCATCACAATCAGATTTTTAATTCTACTTCT 2940
                **** ***   ***** ***::**:.:*:***:           **.  *.*

YJ      TTTTCTCCAGAATAATGTGTGAG------TAGTTCCCAG---------ATAAGGGAATTA 2958
LUCH    ACTCCTCATCAAAATTGGCTGCAGACAAATGGCTTCCAACCTCCTCTCATGAGACCTTCT 3000
        : * ***.: **:*:**  **..      *.* * ***.         **.**. .:* :

YJ      GGGTTCTTATA---------GGGTTTCGCTCATGTGTTGAGCATATAAGAAACCCTTAGT 3009
LUCH    TGAATCTTTTATATTTTTAAGGTTTATTATTATATAAGAAAAACAAAAATGAACCTTTGA 3060
         *.:****:**         ** **:  .* **.*.: .*..* *:**.:.*.****:*:

YJ      ATG---TATTTGTATTTGTAAAATACTTCTATCAAT---AAAATTTCTAATTCCTAAAAC 3063
LUCH    AATCCCCACATGTTCTTGGTCATTTCATTAATCATCGGCTTATATTTTGCTTATTTTCCC 3120
        *:     * :***: *** :.*:*:*:* :****:    ::*::** *..**. *::..*

YJ      CAAAATCCAG-TGACCTGCAGGTCGACTCTAGAGGATCCCCGGGTACCGAG---CTCGAA 3119
LUCH    CTAAATCCTCTTGTTAACTTAGGCGAACAAAAAAAATTAATGGAAATCTTTTTCCCTGAA 3180
        *:******:  **: .:  :.* ***. .:*.*..** .. **.:* * :    *  ***

YJ      TTCGTAATCATGGTCATAGCTGTTTCCTGTGTGAAATTGTTATCCGCTCACAATTCCACA 3179
LUCH    TTCGTAATCATG-TCATAGCTGTTTCCTGTGTGAAATTGTTATCCGCTCACAATTCCACA 3239
        ************ ***********************************************

YJ      CAACATACGAGCCGGAAGCATAAAGTGTAAAGCCTGGGGTGCCTAATGAGTGAGCTAACT 3239
LUCH    CAACATACGAGCCGGAAGCATAAAGTGTAAAGCCTGGGGTGCCTAATGAGTGAGCTAACT 3299
        ************************************************************

YJ      CACATTAATTGCGTTGCGCTCACTGCCCGCTTTCCAGTCGGGAAACCTGTCGTGCCAGCT 3299
LUCH    CACATTAATTGCGTTGCGCTCACTGCCCGCTTTCCAGTCGGGAAACCTGTCGTGCCAGCT 3359
        ************************************************************

YJ      GCATTAATGAATCGGCCAACGCGCGGGGAGAGGCGGTTTGCGTATTGGCTAGAGCAGCTT 3359
LUCH    GCATTAATGAATCGGCCAACGCGCGGGGAGAGGCGGTTTGCGTATTGGCTAGAGCAGCTT 3419
        ************************************************************

YJ      GCCAACATGGTGGAGCACGACACTCTCGTCTACTCCAAGAATATCAAAGATACAGTCTCA 3419
LUCH    GCCAACATGGTGGAGCACGACACTCTCGTCTACTCCAAGAATATCAAAGATACAGTCTCA 3479
        ************************************************************

YJ      GAAGACCAAAGGGCTATTGAGACTTTTCAACAAAGGGTAATATCGGGAAACCTCCTCGGA 3479
LUCH    GAAGACCAAAGGGCTATTGAGACTTTTCAACAAAGGGTAATATCGGGAAACCTCCTCGGA 3539
        ************************************************************

YJ      TTCCATTGCCCAGCTATCTGTCACTTCATCAAAAGGACAGTAGAAAAGGAAGGTGGCACC 3539
LUCH    TTCCATTGCCCAGCTATCTGTCACTTCATCAAAAGGACAGTAGAAAAGGAAGGTGGCACC 3599
        ************************************************************
```

```
YJ              TACAAATGCCATCATTGCGATAAAGGAAAGGCTATCGTTCAAGATGCCTCTGCCGACAGT 3599
LUCH            TACAAATGCCATCATTGCGATAAAGGAAAGGCTATCGTTCAAGATGCCTCTGCCGACAGT 3659
                ************************************************************

YJ              GGTCCCAAAGATGGACCCCCACCCACGAGGAGCATCGTGGAAAAAGAAGACGTTCCAACC 3659
LUCH            GGTCCCAAAGATGGACCCCCACCCACGAGGAGCATCGTGGAAAAAGAAGACGTTCCAACC 3719
                ************************************************************

YJ              ACGTCTTCAAAGCAAGTGGATTGATGTGATAACATGGTGGAGCACGACACTCTCGTCTAC 3719
LUCH            ACGTCTTCAAAGCAAGTGGATTGATGTGATAACATGGTGGAGCACGACACTCTCGTCTAC 3779
                ************************************************************

YJ              TCCAAGAATATCAAAGATACAGTCTCAGAAGACCAAAGGGCTATTGAGACTTTTCAACAA 3779
LUCH            TCCAAGAATATCAAAGATACAGTCTCAGAAGACCAAAGGGCTATTGAGACTTTTCAACAA 3839
                ************************************************************

YJ              AGGGTAATATCGGGAAACCTCCTCGGATTCCATTGCCCAGCTATCTGTCACTTCATCAAA 3839
LUCH            AGGGTAATATCGGGAAACCTCCTCGGATTCCATTGCCCAGCTATCTGTCACTTCATCAAA 3899
                ************************************************************

YJ              AGGACAGTAGAAAAGGAAGGTGGCACCTACAAATGCCATCATTGCGATAAAGGAAAGGCT 3899
LUCH            AGGACAGTAGAAAAGGAAGGTGGCACCTACAAATGCCATCATTGCGATAAAGGAAAGGCT 3959
                ************************************************************

YJ              ATCGTTCAAGATGCCTCTGCCGACAGTGGTCCCAAAGATGGACCCCCACCCACGAGGAGC 3959
LUCH            ATCGTTCAAGATGCCTCTGCCGACAGTGGTCCCAAAGATGGACCCCCACCCACGAGGAGC 4019
                ************************************************************

YJ              ATCGTGGAAAAAGAAGACGTTCCAACCACGTCTTCAAAGCAAGTGGATTGATGTGATATC 4019
LUCH            ATCGTGGAAAAAGAAGACGTTCCAACCACGTCTTCAAAGCAAGTGGATTGATGTGATATC 4079
                ************************************************************

YJ              TCCACTGACGTAAGGGATGACGCACAATCCCACTATCCTTCGCAAGACCCTTCCTCTATA 4079
LUCH            TCCACTGACGTAAGGGATGACGCACAATCCCACTATCCTTCGCAAGACCCTTCCTCTATA 4139
                ************************************************************

YJ              TAAGGAAGTTCATTTCATTTGGAGAGGACACGCTGAAATCACCAGTCTCTCTCTACAAAT 4139
LUCH            TAAGGAAGTTCATTTCATTTGGAGAGGACACGCTGAAATCACCAGTCTCTCTCTACAAAT 4199
                ************************************************************

YJ              CTATCTCTCTCGATTCGCAGATCTGTCGATCGACCATGGGGATTGAACAAGATGGATTGC 4199
LUCH            CTATCTCTCTCGATTCGCAGATCTGTCGATCGACCATGGGGATTGAACAAGATGGATTGC 4259
                ************************************************************

YJ              ACGCAGGTTCTCCGGCCGCTTGGGTGGAGAGGCTATTCGGCTATGACTGGGCACAACAGA 4259
LUCH            ACGCAGGTTCTCCGGCCGCTTGGGTGGAGAGGCTATTCGGCTATGACTGGGCACAACAGA 4319
                ************************************************************

YJ              CAATCGGCTGCTCTGATGCCGCCGTGTTCCGGCTGTCAGCGCAGGGGCGCCCGGTTCTTT 4319
LUCH            CAATCGGCTGCTCTGATGCCGCCGTGTTCCGGCTGTCAGCGCAGGGGCGCCCGGTTCTTT 4379
                ************************************************************

YJ              TTGTCAAGACCGACCTGTCCGGTGCCCTGAATGAACTCCAGGACGAGGCAGCGCGGCTAT 4379
LUCH            TTGTCAAGACCGACCTGTCCGGTGCCCTGAATGAACTCCAGGACGAGGCAGCGCGGCTAT 4439
                ************************************************************

YJ              CGTGGCTGGCCACGACGGGCGTTCCTTGCGCAGCTGTGCTCGACGTTGTCACTGAAGCGG 4439
LUCH            CGTGGCTGGCCACGACGGGCGTTCCTTGCGCAGCTGTGCTCGACGTTGTCACTGAAGCGG 4499
                ************************************************************

YJ              GAAGGGACTGGCTGCTATTGGGCGAAGTGCCGGGGCAGGATCTCCTGTCATCTCACCTTG 4499
```

```
LUCH            GAAGGGACTGGCTGCTATTGGGCGAAGTGCCGGGGCAGGATCTCCTGTCATCTCACCTTG 4559
                ************************************************************

YJ              CTCCTGCCGAGAAAGTATCCATCATGGCTGATGCAATGCGGCGGCTGCATACGCTTGATC 4559
LUCH            CTCCTGCCGAGAAAGTATCCATCATGGCTGATGCAATGCGGCGGCTGCATACGCTTGATC 4619
                ************************************************************

YJ              CGGCTACCTGCCCATTCGACCACCAAGCGAAACATCGCATCGAGCGAGCACGTACTCGGA 4619
LUCH            CGGCTACCTGCCCATTCGACCACCAAGCGAAACATCGCATCGAGCGAGCACGTACTCGGA 4679
                ************************************************************

YJ              TGGAAGCCGGTCTTGTCGATCAGGATGATCTGGACGAAGAGCATCAGGGGCTCGCGCCAG 4679
LUCH            TGGAAGCCGGTCTTGTCGATCAGGATGATCTGGACGAAGAGCATCAGGGGCTCGCGCCAG 4739
                ************************************************************

YJ              CCGAACTGTTCGCCAGGCTCAAGGCGCGCATGCCCGACGGCGAGGATCTCGTCGTGACAC 4739
LUCH            CCGAACTGTTCGCCAGGCTCAAGGCGCGCATGCCCGACGGCGAGGATCTCGTCGTGACAC 4799
                ************************************************************

YJ              ATGGCGATGCCTGCTTGCCGAATATCATGGTGGAAAATGGCCGCTTTTCTGGATTCATCG 4799
LUCH            ATGGCGATGCCTGCTTGCCGAATATCATGGTGGAAAATGGCCGCTTTTCTGGATTCATCG 4859
                ************************************************************

YJ              ACTGTGGCCGGCTGGGTGTGGCGGACCGCTATCAGGACATAGCGTTGGCTACCCGTGATA 4859
LUCH            ACTGTGGCCGGCTGGGTGTGGCGGACCGCTATCAGGACATAGCGTTGGCTACCCGTGATA 4919
                ************************************************************

YJ              TTGCTGAAGAGCTTGGCGGCGAATGGGCTGACCGCTTCCTCGTGCTTTACGGTATCGCCG 4919
LUCH            TTGCTGAAGAGCTTGGCGGCGAATGGGCTGACCGCTTCCTCGTGCTTTACGGTATCGCCG 4979
                ************************************************************

YJ              CTCCCGATTCGCAGCGCATCGCCTTCTATCGCCTTCTTGACGAGTTCTTCTGAGCGGGAC 4979
LUCH            CTCCCGATTCGCAGCGCATCGCCTTCTATCGCCTTCTTGACGAGTTCTTCTGAGCGGGAC 5039
                ************************************************************

YJ              TCTGGGGTTCGGATCGATCCTCTAGCTAGAGTCGATCGACATCGAGTTTCTCCATAATAA 5039
LUCH            TCTGGGGTTCGGATCGATCCTCTAGCTAGAGTCGATCGACATCGAGTTTCTCCATAATAA 5099
                ************************************************************

YJ              TGTGTGAGTAGTTCCCAGATAAGGGAATTAGGGTTCTTATAGGGTTTCGCTCACGTGTTG 5099
LUCH            TGTGTGAGTAGTTCCCAGATAAGGGAATTAGGGTTCTTATAGGGTTTCGCTCACGTGTTG 5159
                ************************************************************

YJ              AGCATATAAGAAACCCTTAGTATGTATTTGTATTTGTAAAATACTTCTATCAATAAAATT 5159
LUCH            AGCATATAAGAAACCCTTAGTATGTATTTGTATTTGTAAAATACTTCTATCAATAAAATT 5219
                ************************************************************

YJ              TCTAATTCCTAAAACCAAAATCCAGTACTAAAATCCAGATCACCTAAAGTCCCTATAGAT 5219
LUCH            TCTAATTCCTAAAACCAAAATCCAGTACTAAAATCCAGATCACCTAAAGTCCCTATAGAT 5279
                ************************************************************

YJ              CCCCCGAATTAATTCGGCGTTAATTCAGTACATTAAAAACGTCCGCAATGTGTTATTAAG 5279
LUCH            CCCCCGAATTAATTCGGCGTTAATTCAGTACATTAAAAACGTCCGCAATGTGTTATTAAG 5339
                ************************************************************

YJ              TTGTCTAAG 5288
LUCH            TTGTCTAAG 5348
                *********
```
